# Fostering productive conversations in a Kenyan tertiary intensive care unit: lessons learnt

**DOI:** 10.11604/pamj.2019.34.104.20526

**Published:** 2019-10-21

**Authors:** Sayed Karar Ali, Jamila Nambafu, Feroza Daroowalla

**Affiliations:** 1 Department of Internal Medicine, The Aga Khan University, Nairobi, Kenya; 2 Department of Medicine, University of Central Florida, College of Medicine, Orlando, Florida, USA

**Keywords:** Communication, intensive care unit, sub-Saharan Africa

## Abstract

Effective communication remains key in delivery of comprehensive care to patient especially in the intensive care unit (ICU) setting. However, many providers, for various reasons, struggle with the art of effective communication adversely affecting relationship with patients and their families. Little is known or has been published about effective communication in ICUs within sub-Saharan Africa.

## Introduction

Effective communication remains key in delivery of care to the most critically ill being cared for in the intensive care unit (ICU). Despite the importance of effective communication, providers in the ICU struggle with delivery of information, often affecting overall care including the relationship with the patient and family members. Many strategies have been proposed to enhance effective communication in the ICU. However, little is known or has been published about effective communication in sub-Saharan Africa. We present a case in our ICU where better communication strategies would have greatly influenced the overall patient outcomes and improved family satisfaction.

## Patient and observation

A 51-year-old non-compliant hypertensive female was admitted to an outside institution with headaches and neck stiffness for about one month. She was diagnosed with pyogenic meningitis complicated by multiple generalized tonic-clonic seizures. She was subsequently transferred to our institution for further care where a CT brain revealed a ruptured 4 by 3 mm anterior communicating artery saccular aneurysm with infarction of the anterior cerebral artery territory. The neurosurgery team performed clipping of the artery, which she tolerated well. However, a few days post-surgery, she had a sudden degradation in her level of consciousness, requiring intubation to protect her airway. A repeat CT brain showed increasing hydrocephalus necessitating VP Shunt. Her hospital courses was complicated by several cerebrospinal fluid infections including multi-drug resistant *pseudomonas* and *acitenobacter* which required multiple broad-spectrum antibiotics, removal of the VP shunt and placement of an extra ventricular drain. She also had various electrolyte abnormalities and continued to have seizures that eventually required barbiturates for control. She has had an ICU stay of six months and remains intermittently ventilator dependent, bed-bound and unable to follow simple commands or have meaningful communication. Upon further discussion with the family, they revealed that they had not received relevant information from the ICU team regarding goals of care and choices in medical management that could have influenced their earlier medical decisions. They voiced that a lack of honest and frequent communication from the medical team had left them questioning the medical treatment provided to their loved one and somewhat unprepared for the future.

## Discussion

The intensive care unit (ICU) tends to the care of the most critical patients with dynamic medical needs. Communication about the medical conditions and decision-making about goals of care falls to the patient's family and surrogate decision makers. Medical providers in the ICU grapple with communication of medically and morally complex issues and often lack the training to adequately maneuver such conversations [1]. Adequate, sensitive, timely and effective communication between families and the clinical team, especially at the end of life, consists of succinct, regular and easy to process updates [2]. Engaging in these discussions is complicated by the varying expectations of the family and divergent views from those of the ICU team [3]. Poor communication has also been shown to adversely impact the patient outcomes. Furthermore, inadequate communication may contribute to various psychological problems in family members face including depression, sleep disturbances, post-traumatic stress disorder and anxiety [3]. Similar to the case above, many families feel that important information is not adequately communicated and this often results in unhealthy untrusting relationships between the families and medical providers [3]. It has been strongly advocated that training in communications should be competency based similar to any other ICU procedure [4]. However, this is seldom practiced and the role of communication is not fully appreciated even by the most experienced provider. Furthermore, there is little research on how to effectively plan, teach and foster effective communication strategies in the unique cultural milieu of sub Saharan Africa [5]. Some Barriers to effective communication with families are common to all parts of the world. One such barrier is the lack of training for medical providers to have difficult discussions with family members. These discussions can often be emotionally charged resulting in emotional overload and eventual burnout for the medical team. Many provider also feel that family meetings are lengthy and this is a deterrent for those with time constraints and who need to prioritize clinical care for critically ill patients. In addition, many medical providers struggle with prognostication and feel unqualified to directly answer questions posed by family members [6]. Many providers are not trained and find it challenging to roll out these difficult conversations in a framework easy to comprehend for the family. In addition, many medical providers are not trained to pick and adequately address emotional cues from family members.

Patient and family factors can also impede effective communication and decision making [6]. Patient and family denial of diagnosis and prognosis; anxiety surrounding uncertainty of course and outcome are examples of these. Expectation of patients and their families for recovery and positive outcomes differ from those of the ICU team especially around end of life care and this can often results in poor shared decision-making. Many methods have been looked at to improve the process of sharing information with family members. Allen and colleagues found that incorporating families into the daily patient rounds improved communication and satisfaction. Families had a chance to be involved in the medical care and ask questions. Encompassing families into the daily rounds also helped shift time away from lengthy family meetings [7]. Others have suggested that nurses, who spend a significant time with the patient and families, may be utilized as a resource to ensure that families understand the day-to-day activities in the ICU in simple non-medical terms. This strategy has been shown to enhance the flow of information and improve the overall communication process with family members [8]. A strategy of recruiting skilled communication experts to facilitate conversation between medical team and families, adapt conversation to family needs and assist with conflict resolution has been shown to decrease depression, anxiety and PTSD in family members six month later [9]. A palliative care team has the training to foster conversations with families and have been shown to improve communication, care and cost in the ICU [10]. Family meetings consisting of family members and the medical team including physicians, nurses and other key providers, remain a key method of communication with families in the ICU. Many families reported satisfaction when family conferences were held regularly and when physicians supported decisions made by families in regards to end of life; and provided assurances of continued high quality care and patient comfort [11,12]. There is a need to develop and deliver communication skills, education and assessments relevant to providers and patients in Sub-Saharan Africa ICU settings. Empathetic, tactful and skilled communication where a medical provider is able to listen and respond to emotion and share outcomes have been shown to be markers of quality care and should be considered as competencies to help train ICU physicians and other ICU clinicians [13].

## Conclusion

In the case discussed above, earlier and more frequent skillful communication could have helped the family with critical decision-making and to better appreciate the quality of care provided to their loved ones. Any of the above strategies may have improved patient's outcome and family satisfaction. There remain a critical need for research in determining the utility of various communication strategies, including the regular involvement of palliative care trained clinicians in seriously ill and ICU patients in sub-Saharan Africa.

## Competing interests

The authors declare no competing interests.
